# Culturable endophytic fungi community structure isolated from *Codonopsis pilosula* roots and effect of season and geographic location on their structures

**DOI:** 10.1186/s12866-023-02848-3

**Published:** 2023-05-15

**Authors:** Lili Fan, Yuanli Li, Xiaoli Wang, Feifan Leng, Shaowei Li, Ning Zhu, Kai Chen, Yonggang Wang

**Affiliations:** 1 School of Life Science and Engineering, Lanzhou University of Technology, Lanzhou, 730050 China; 2 Lanzhou Institute of Husbandry and Pharmaceutical Sciences, Chinese Academy of Agricultural Sciences, Lanzhou, 730050 China; 3 Key Laboratory of Ecosystem Network Observation and Modeling, Institute of Geographic Sciences and Natural Resources Research, Chinese Academy of Sciences, Beijing, 100101 China

**Keywords:** *Codonopsis pilosula*, Endophytic fungi diversity, Soil characterization, Temporal and spatial variation, Geographical location, Correlation analysis

## Abstract

**Background:**

Rhizosphere soil physicochemical, endophytic fungi have an important role in plant growth. A large number of endophytic fungi play an indispensable role in promoting plant growth and development, and they can provide protection for host plants by producing a variety of secondary metabolites to resist and inhibit plant pathogens. Due to the terrain of Gansu province is north–south and longitudinal, different climatic conditions, altitude, terrain and growth environment will affect the growth of *Codonopsis pilosula*, and the changes in these environmental factors directly affect the quality and yield of *C. pilosula* in different production areas. However, In *C. pilosula*, the connection between soil nutrients, spatiotemporal variation and the community structure of endophytic fungi isolated from *C. pilosula* roots has not been well studied.

**Results:**

Seven hundred six strains of endophytic fungi were obtained using tissue isolation and the hyphaend-purification method from *C. pilosula* roots that picked at all seasons and six districts (Huichuan, HC; Longxi, LX; Zhangxian, ZX; Minxian, MX; Weiyuan, WY; and Lintao, LT) in Gansu Province, China. *Fusarium* sp*.* (205 strains, 29.04%), *Aspergillus* sp*.* (196 strains, 27.76%), *Alternaria* sp*.* (73 strains, 10.34%), *Penicillium* sp*.* (58 strains, 8.22%) and *Plectosphaerella* sp*.* (56 strains, 7.93%) were the dominant genus. The species composition differed from temporal and spatial distribution (Autumn and Winter were higher than Spring and Summer, MX and LT had the highest similarity, HC and LT had the lowest). physical and chemical of soil like Electroconductibility (EC), Total nitrogen (TN), Catalase (CAT), Urease (URE) and Sucrase (SUC) had significant effects on agronomic traits of *C. pilosula* (*P* < 0.05). AK (Spring and Summer), TN (Autumn) and altitude (Winter) are the main driving factors for the change of endophytic fungal community. Moreover, geographic location (such as altitude, latitude and longitude) also has effects on the diversity of endophytic fungi.

**Conclusions:**

These results suggested that soil nutrients and enzyme, seasonal variation and geographical locations have an impact on shaping the community structure of culturable endophytic fungi in the roots of *C. pilosula* and its root traits. This suggests that climatic conditions may play a driving role in the growth and development of *C. pilosula*.

**Supplementary Information:**

The online version contains supplementary material available at 10.1186/s12866-023-02848-3.

## Introduction

*Codonopsis pilosula,* a traditional medicinal plant, can not only enhance the immune and gastrointestinal functions of the body, but also nourish the spleen and lungs [1]. Located in arid and semi-arid areas, Gansu Province of China is the main production area of *C. pilosula* [2]. soil nutrition and endophytic fungal community structure play an important role in shaping the quality of medicinal plants [3–5] However, there are relatively few reports on the role of endophytic fungi in plant root growth of *C. pilosula.*

Soil microbes regulate soil nutrient cycling processes and plant productivity, which are important soil health indicators [6–8]. Microbes drive many processes required for plant growth and health. Studies have shown that the effects of plant and soil microbial diversity on the multi-functionality of ecosystems are synergistic and complementary [9–12]. Terrestrial plants contain a variety of microbes colonized roots, which affect the health and growth of plants in a beneficial, harmful or neutral way [13]. Studies have shown that metabolism and accumulation of active components in medicinal plants are significantly affected by plant varieties, soil nutrients, endosymbiotic microorganisms and environmental factors (such as climate factors, agronomic measures, harvesting, processing and storage methods, etc.) [14, 15]. Among all factors as mentioned earlier, Endophytic fungi play essential roles in the production of plant bioactive metabolites [16–18]. Endophytic fungi can regulate host plants to resist biotic and abiotic stresses [19–21], Studies have found that dark septal endophytic fungi (DSE) usually exist in the roots of trees (such as coniferous forests, poplars, etc.) and herbaceous plants in saline-alkali soils of tidal flats, which can improve plant growth and stress resistance. Especially in extreme environments, its abundance is often higher than mycorrhizal fungi. Arbuscular mycorrhizal fungi inhabit the rhizosphere of medicinal plants, promote the growth of medicinal plants and accelerate the accumulation of medicinal components [22]. In addition, salt stress affects growth and characteristics of medicinal plants [23]. These research results indicate that endophytic fungi play an important part in the growth and development and quality shaping of medicinal plants.

To reveal the seasonal and geographical effects of culturable endophytic fungi in *C. pilosula* root on plant root growth, we isolated and purified endophytic fungi from *C*. *pilosula* by pure culture technology, measured soil nutrients and enzyme activities, and clarified the effects of soil nutrients, seasonal changes and geographical locations on the diversity and structure of culturable endophytic fungi in the root of *C. pilosula* in the process of temporal and spatial change.

## Materials and methods

### Plant and soil samples collection

Plant samples and soil samples including rhizosphere soil attached to the root surface thickness of 2 cm and non-rhizosphere soil without culture any plants in the same area were collected from six main producing areas (LX, ZX, WY MX, LT and HC) in Gansu, China. Healthy and fresh *C. pilosula* roots were collected in sterile plastic bags, labeled and stored in the laboratory at 4 ℃ for 48 h. The basic situation of the sampling sites of *C. pilosula* and collection times were listed in the Supplementary text.

The root length (RL) and diameter (RD) of *C. pilosula* were measured with vernier caliper, and the root weight (RW) was measured with gravimetric method.

### Isolation and purification of endophytic fungi from *C. pilosula* roots

*C. pilosula* samples were treated by surface disinfection [24]. These treated roots were cut into approximate 5 mm pieces and placed on six fungal culture media (PDA Medium, Czapek-Dox Agar Medium, Sabourand’s Agar Medium, Rose Bengal Medium, MS Medium, and CYM Medium) with streptomycin sulphate (50 mg/L) and ampicillin (100 μg/mL) to inhibit bacteria. 100 μl of sterile water washed last time with surface disinfection is applied to the above six fungal culture Medium as a control, after 7 days of incubation, observe whether there are fungi growing, if there is, it proves that the disinfection is not thorough, and the experiment needs to be re-carried out; Conversely, it turns out that the growing colony are endophytic fungi. The pure culture was obtained according to the method of Qiu et al. [25].

### Identification of endophytic fungi from *C. pilosula* roots

According to the macroscopic colony characteristics, microscopic individual morphological characteristics and some biological characteristics, the isolated endophytic fungi of *C. pilosula* were morphologically classified by the method of lactic acid phenol and identified. In detail, referring to the fungal identification manual [26], all strains were first identified based on the morphological characteristics of the colony and hypha or spores (BM2000D, Nanjing Jiangnan Yongxin Optical Co., Ltd., Nanjing, China). Meanwhile, the molecular biological identification was carried out to confirm further its species, modified CTAB method was used to extract DNA. All strains ITS sequences information was submitted to NCBI by Bankit tool (https://www.ncbi.nlm.nih.gov/).

### Endophytic fungal diversity analysis

The abundance and diversity of endophytic fungi in the root of *C. pilosula* were analyzed at the genus level. Isolation rate (IR, %) was used to calculate the abundance of endophytic fungi in plant tissues. Relative frequency (Isolation frequency, IF) is used to compare the dominant flora of endophytic fungi [27]. The Shannon-Weiner diversity index was used to analyze the diversity level of endophytic fungi populations. The Pielou uniformity index is used to analyze the uniformity of the bacterial population distribution [27]. The Chao1 index was used to calculate the community abundance, the Coverage sequencing depth index was used to calculate the separation depth, the Pielou uniformity index was used to calculate the uniformity of the flora, and the RDA redundancy analysis method was used to analyze the correlation between the diversity of endophytic fungi in the roots of *C. pilosula* and environmental factors. All calculation formulas are shown in the Supplementary text.

### Determination of soil nutrients and enzymes

The soil physical and chemical properties were determined according to the method described by Guan [28]. In short, the soil pH was determined with a pH meter (HI 98,128, HANNA instruments, USA). Drying weighing is used to determine soil moisture content. Flame photometric (Z5000, Hitachi, Japan) was used to measure total K after the reaction between soil and sodium hydroxide fusion, and the method also was used to determine available K [29]. Kjeldahl method was used to determine total nitrogen. Soil available nitrogen was determined by alkaline hydrolysis diffusion method. Total phosphorus was determined by sodium hydroxide melting method, then colorimetric analysis was carried out, and available phosphorus was determined by molybdenum antimony colorimetry. The electrical conductivity (EC) of soil was measured by the electrode method. The determination of soil organic matter (SOM) was conducted by potassium dichromate volumetric method.

According to the potassium permanganate titration method [28], 2.000 g of soil sample was weighted into an Erlenmeyer flask, and the catalase (CAT) activity was determined. The urease (URE) activity was measured according to the indophenol colorimetric method [28]. The invertase (SUC) was determined according to the 3,5-dinitrosalicylic acid colorimetric method [28], and expressed as the number of mg of glucose released by 1 g of soil in 24 h. The protease (PRO) activity was analyzed according to the modified ninhydrin colorimetric method [30].

### Statistical analysis

The data were analyzed and processed by Microsoft Excel 2007, SPSS19.0 (IBM), R (v3.6.2, v4.0.4): ggplot2, vegan, permute, lattice, circlize, AMOS 26.0 (IBM) and Origin8.0 (Origin Lab). Mantel Test was performed on Tutools platform (http://www.cloudtutu.com). the difference of soil physical and chemical properties in four seasons of six main producing areas of *C. pilosula* in Gansu was tested by single factor ANOVA (α = 0.05). The redundancy analysis of soil physical and chemical factors, temperature and altitude was analyzed by Mantel Test. All data are mean ± standard error. Each group of experiments was repeated three times.

## Results

### Isolation rate and classification of endophytic fungi from* C. pilosula roots*

**Fig. 1:**
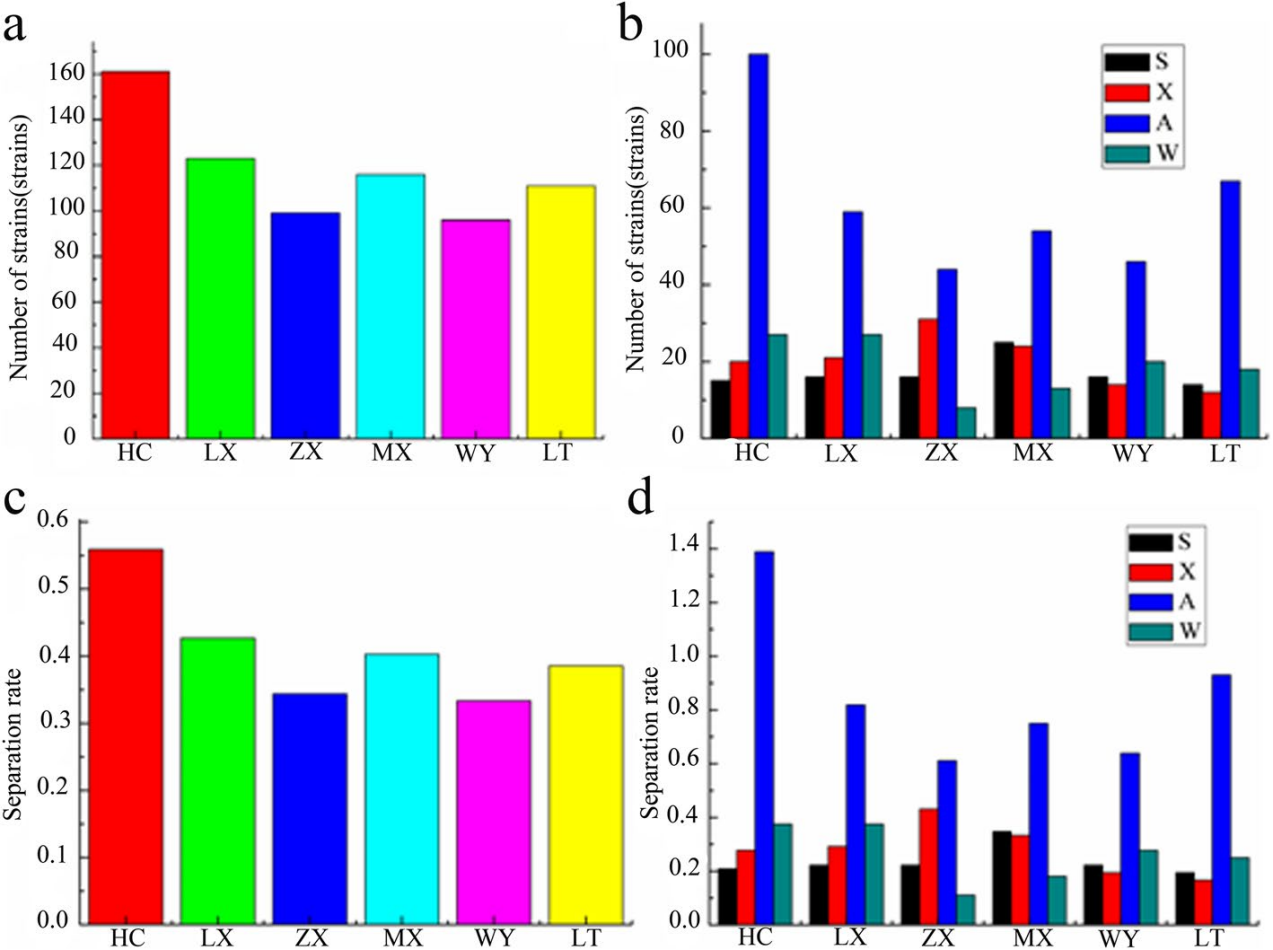
Isolation number and isolation rate of endophytic fungi from *C. pilosula* root in six areas and four seasons. **a** Number of isolated strains in six areas. **b** Number of isolated strains in all seasons in six areas. **c** Separation rate in six areas. **d** Separation rate in all seasons in six regions. In terms of region, HC has the highest number of separated plants and separation rate, WY is the lowest, and in terms of season, autumn has the highest number of isolated plants and separation rate

After 7 days of plate culture in the control group of six kinds of fungal isolation medium, no colonies grew on the plate, which proved that the root surface of *C. pilosula* was thoroughly disinfected, and the fungi obtained by the experiment were all endophytic fungi. 706 strains of fungi were isolated from 72 *C. pilosula* root samples in four seasons in the six main production areas of Gansu Province. Among them, 204 representative strains were selected for morphological observation and recording, and 20 strains were selected from 204 strains of endophytic fungi that were morphologically different from other strains for detailed description and molecular identification. Colony Morphology and Micrographics of Endophytic Fungi Cultured from the Root of 20 *C. pilosula* in sFig. 1. The number and rate of strains separated in six regions and all seasons were calculated as shown in Fig. 1. 162 strains were isolated from HC, with the isolation rate of 55.90% (15 strains in spring, 20.83%; 20 strains in summer, 27.78%; 100 strains in autumn, 138.89%; 27 strains in winter, 37.50%). 123 strains were isolated from LX, and the isolation rate was 42.71% (16 strains in spring, 22.22%; 21 strains in summer, 29.17%; 59 strains in autumn, 81.94%; 27 strains in winter, 37.50%). 99 strains were isolated from ZX, and the isolation rate was 34.38% (16 strains in spring, 22.22%; 31 strains in summer, 43.06%; 44 strains in autumn, 61.11%; 8 strains in winter, 11.11%), 116 strains were isolated from MX, and the isolation rate was 40.28% (25 strains in spring, 34.72%; 24 strains in summer, 33.33%; 54 strains in autumn, 75.00%; 13 strains in winter, 18.06%), 96 strains were isolated from WY, and the isolation rate was 33.33% (16 strains in spring, 22.22%; 14 strains in summer, 19.44%; 46 strains in autumn, 63.89%; 20 strains in winter, 27.78%). 111 strains were isolated from LT, and the isolation rate was 38.54% (14 strains in spring, 19.44%; 12 strains in summer, 16.67%; 67 strains in autumn, 93.06%; 18 strains in winter, 25.00%). In regions, HC area had the most isolated strains of 162 and the highest isolation rate of 55.90%, followed by LX and WY. In terms of seasons, the most numbers of strain were isolated in autumn, and the isolation rate of 52.41% was the highest.

**Fig. 2:**
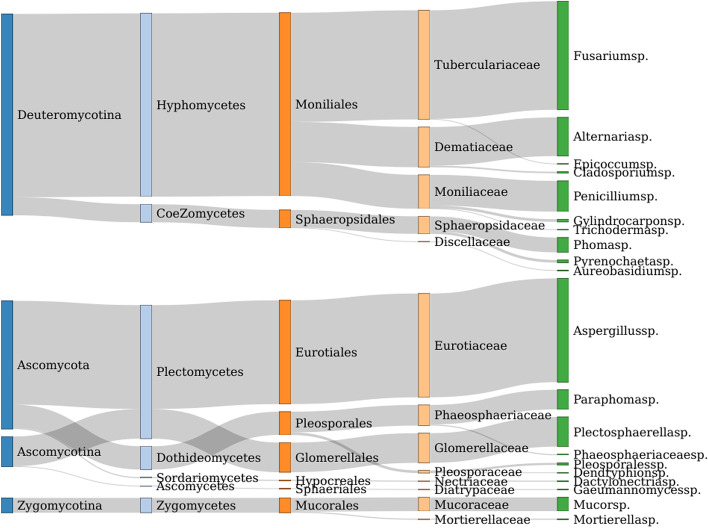
Species taxonomic relationship. The endophytic fungi in the roots of *C. pilosula* can be divided into 4 subphylums, 7 classes, 8 orders, 14 species and 20 genera. The dominant species are as follows from large to small according to the classification level: Deuteromycotina, Hyphomycetes, Moniliales, Tuberculariaceae and *Fusarium sp*

As can be seen from Fig. 2 that endophytic fungi in the root of *C. pilosula* have rich diversity characteristics in quantity and species. The morphology and molecular biology identification results were combined and found that 706 endophytic fungi belonged to 3 Phyllum, 7 classes, 8 orders, 14 families, 20 genera. deuteromycetina is the dominant phylum in the classification level of the subphylum. There were 380 strains in this subphylum, accounting for the proportion of the total number of strains. That is, the relative frequency was 53.83%. Moniliales (346 strains, 49.01%), pleosporales (45 strains, 6.41%) and sphaeropsidales (34 strains, 4.82%) were the dominant orders. *Fusarium* sp. (205 strains, 29.04%), *Aspergillus* sp. (196 strains, 27.76%), *Alternaria* sp. (73 strains, 10.34%), *Penicillium* sp. (58 strains, 8.22%) and *plectosphaerella* sp. (56 strains, 7.93%) were the dominant genera.

### Alpha diversity of endophytic fungi from *C. pilosula* roots in different plant locations at all seasons

**Fig. 3:**
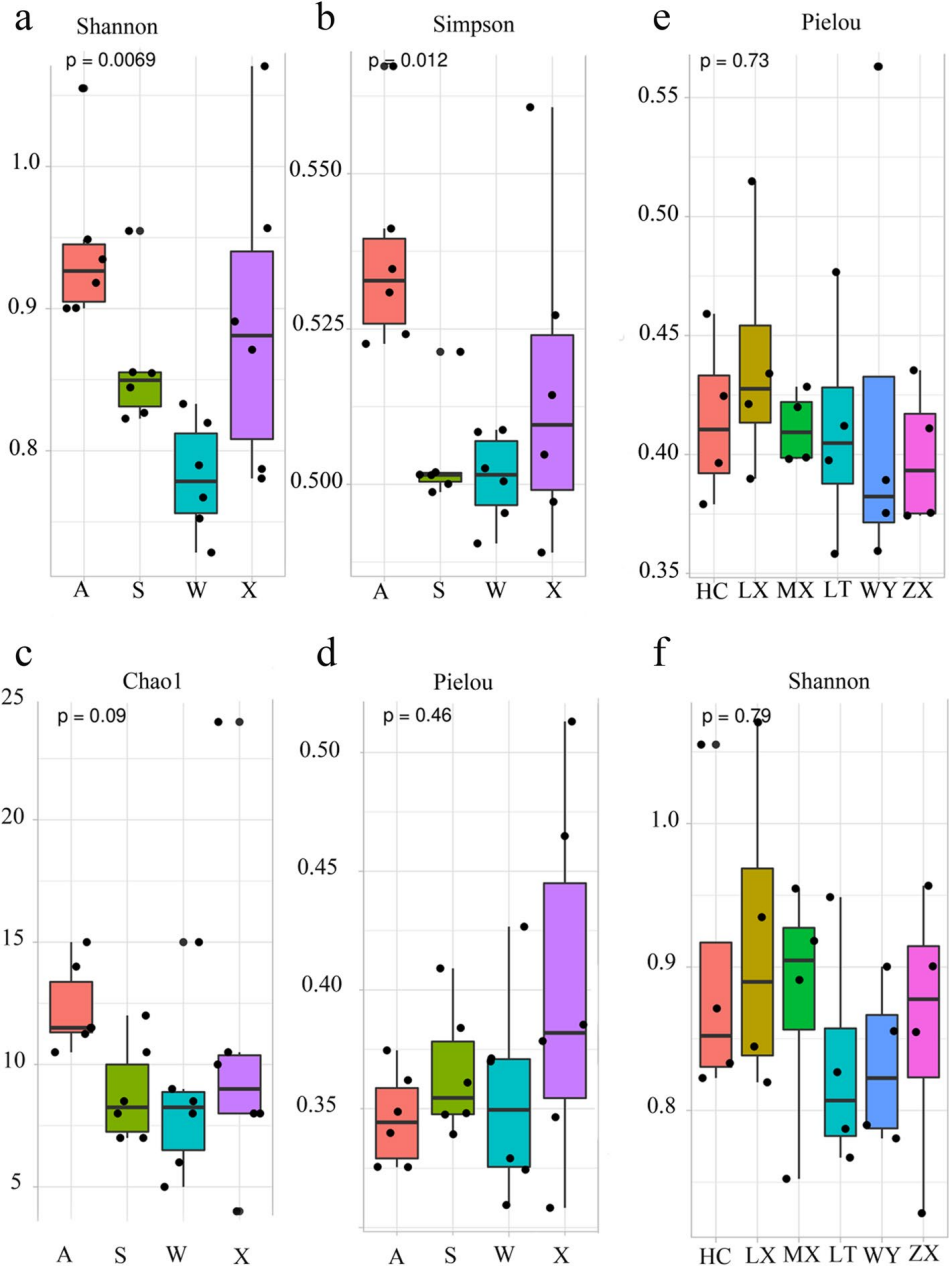
Alpha diversity index under different temporal and spatial conditions (**a**) Alpha diversity index in all seasons. Shannon and Simpson’s index had significant differences (*P* < 0.05). **b** Alpha diversity index in six regions. Shannon and Pielou index had no significant difference (*P* > 0.05)

As can be seen from the Fig. 3, there are significant differences in Shannon–Wiener index, Chao1 index and Pielou index in the six main producing areas of *C. pilosula* in Gansu Province. Shannon–Wiener index and Simpson index showed that HC area community had the highest diversity (H = 3.07) and LX area the lowest (H = 2.33). Chao1 index indicated that HC area community had the highest abundance (29.00) and LX area the lowest (10.00). Pielou index suggested that ZX area has the highest value of fungal community (1.23) and LT area the lowest (0.97). The difference of alpha diversity index in each season was significant (*P* < 0.05), and the contrast of alpha diversity index in six regions was not significant (*P* > 0.05), implying that the species diversity of culturable microorganisms in the roots of *C. pilosula* was affected by seasonal changes.

### Beta diversity analysis of endophytic fungi from *C. pilosula* roots in different plant locations at all seasons

**Fig. 4:**
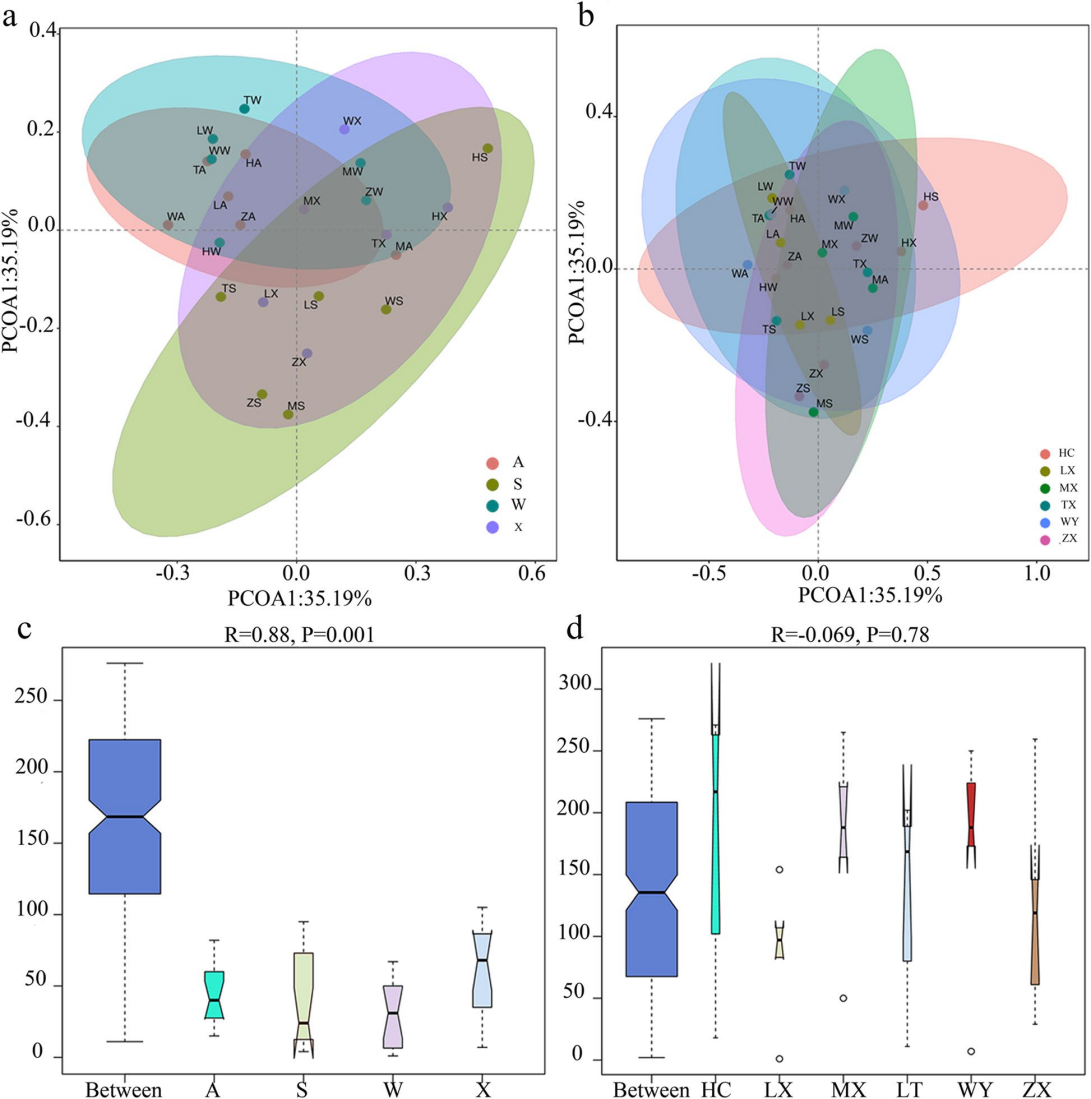
PCoA and ANOSIM analysis based on Bray–Curtis algorithm of samples in 4 seasons and 6 regions. The confidence ellipse represents that 95% of the samples in the group may fall in it. ANOSIM analysis based on Bray–Curtis algorithm used to test whether the difference between groups is more significant than the difference within groups. Different colors represent different groups. **a** Under different seasonal grouping conditions PCoA. **b** Under different regional grouping conditions PCoA. **c** β diversity under different seasonal grouping conditions. **d** β diversity under different regional grouping conditions

PCoA analysis showed that the samples in spring and summer, autumn and winter were clustered into one group respectively without time and space conditions (Fig. 4 a, b), indicating that the roots of *C. pilosula* production areas in spring and summer have a more similar cultivable endophytic fungal community structure, and autumn and winter are closer. At the same time, it also suggested that the differences in the community structure of cultivable endophytic fungi in the roots of *C. pilosula* in spring, summer, autumn and winter were more prominent. For different seasons, central coordinate 1 (PCoA1) and principal coordinate 2 (PCoA2) explained 57.91% of the total variation, which can be identified as the primary source of interpretation. The spring, summer, autumn, and winter samples were separated along the first axis, indicating that the most variation of the samples comes from different time scales (the contribution rate of PCoA1 was 35.19%). The second principal coordinate analysis showed that the random variation of sample repeats within the group can explain 22.72% of the environmental heterogeneity. For different areas, the distribution of samples from the six regions was relatively concentrated, indicating that the roots of *C. pilosula* in 6 areas had a similar community structure of cultivable endophytic fungi. Principal coordinate 1 (PCoA1) and principal coordinate 2 (PCoA2) explained 57.91% of the total variation. Along the first axis and the second axis, the samples from the six main producing areas of *C. pilosula* were not clearly distinguished, indicating that the most variation of the samples came from space scale.

Anosim (Analysis of similarities) test is used in combination with PCoA to determine whether the grouping is meaningful (Fig. 4c, d). The figure reflects the space within and between the sample groups in different regions and different seasons (distances was expressed in Ranks). As can be seen from the Fig. 4 that the distance between groups in the four seasons is greater than the distance within groups, and the difference is significant (*P* = 0.001), indicating that the community structure of cultivable endophytic fungi was various in different groups.

### Species composition of endophytic fungi from *C. pilosula* roots in different plant locations at all seasons

**Fig. 5:**
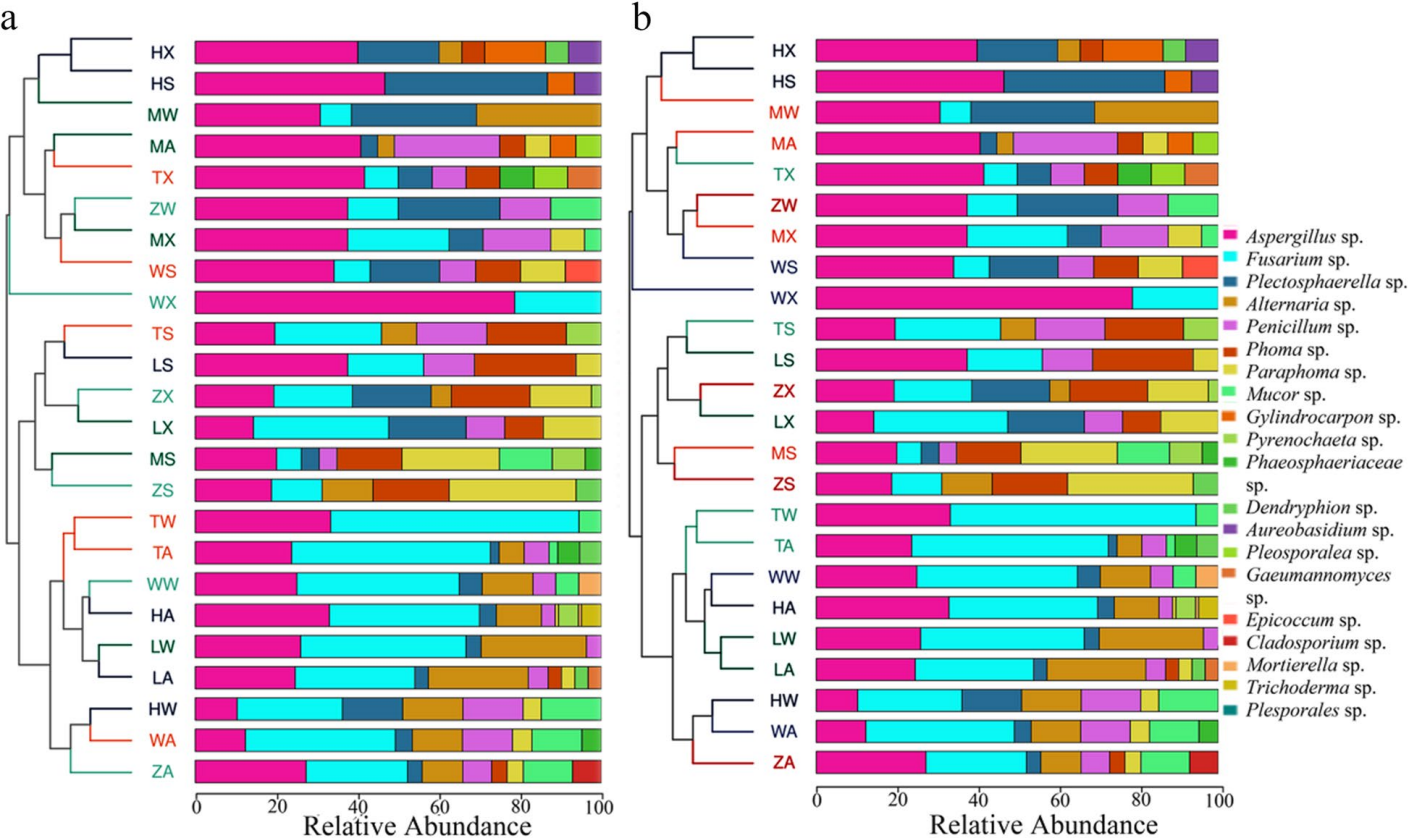
Cluster tree of culturable endophytic fungi can be in the root of *C.pilosula.*(**a** Species composition and clustering conditions under different seasons. **b** Species composition and clustering conditions under different regions

There is a certain degree of similarity and specificity in the species distribution of fungal communities under different temporal and spatial conditions (Fig. 5). Species abundance clustering results showed that the samples in spring and summer, Autumn and winter were in the same large branch, which further implied that the roots of different *C. pilosula* roots in spring and summer were more similar in the community structure of cultivable endophytic fungi in all the sample groups, Autumn and winter were more similar. To learn more about the species composition of each sample under other time and space conditions, the stacked graph shown in the figure showed the top 20 genera in the abundance value of different regions in each season for comparative analysis. It can be seen from the figure that the top 20 abundance species in all samples are *Aspergillus* sp*.*, *Fusarium* sp., *Plectosphaerella* sp*.*, *Alternari*a sp*.*, *Paraphoma* sp*.*, *Penicillium* sp*.*, *Trichoderma* sp*., Aureobasidium* sp*., Cladosporium* sp*., Dendryphion* sp*., Epicoccum* sp*., Gaeumannomyces* sp*., phaeosphaeriaceae* sp*., Mortierella* sp*., Plesporales* sp*., Gylindrocarpon* sp*., Mucor* sp*., Phoma* sp*., Pyrenochaeta* sp. and *Pleosporalea* sp*.* Among them, *Paraphoma* sp*.* and *Phoma* sp*.* had higher abundance in all samples in spring and summer. *Aspergillus* sp*.* and *Fusarium* sp*.* had higher abundance in all samples in autumn and winter.

**Fig. 6:**
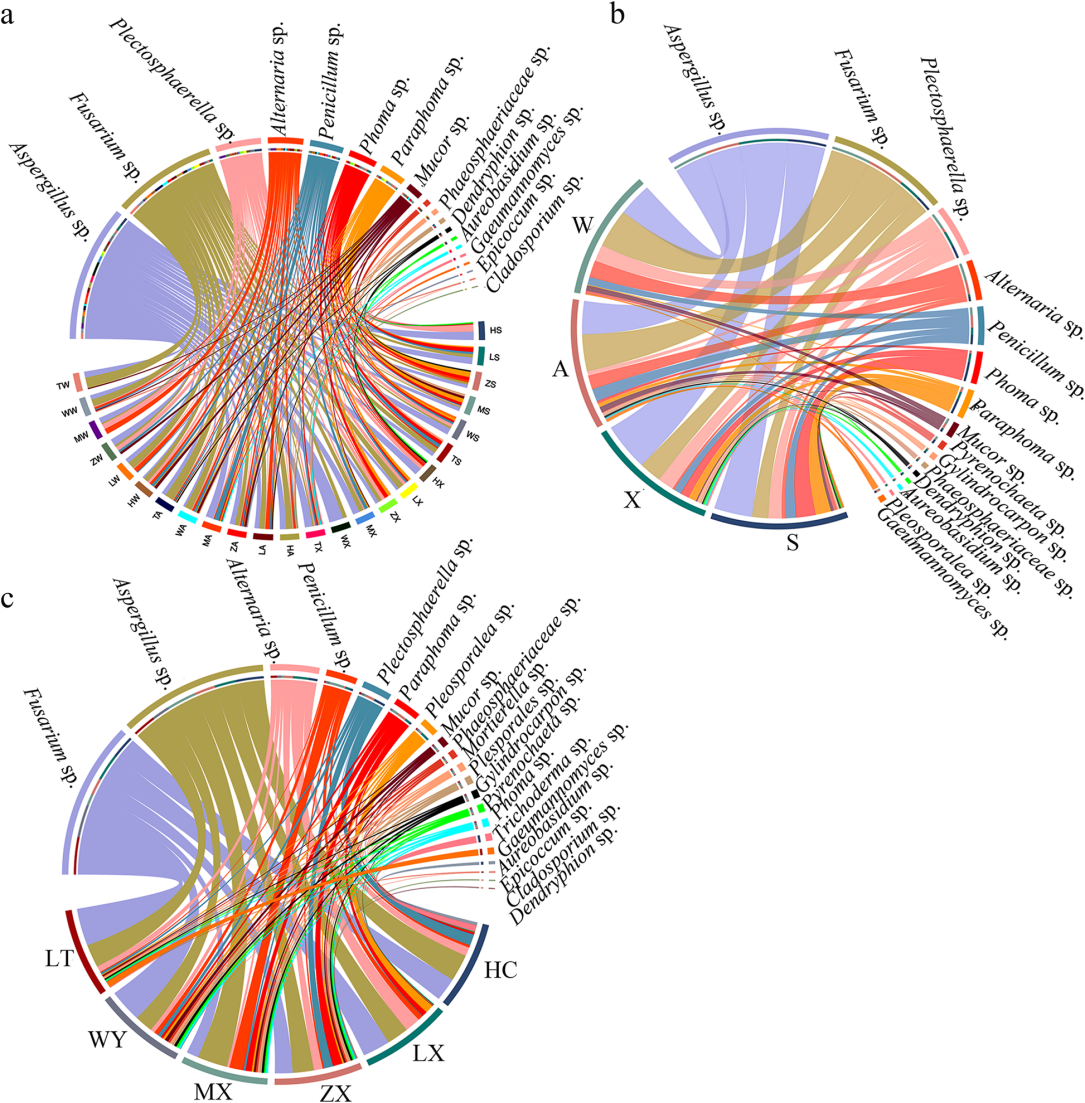
Species composition chord diagram. **a** Species chord diagrams in different seasons and areas. **b** Species chord diagram under different seasons grouping conditions. **c** Species chord diagram under different regions grouping conditions. The species abundance is expressed as a percentage. The line indicates that the species is present in the sample. The thicker the line, the more abundant the species

Different seasonal and geographical changes shape different fungal community composition structures. To observe the influence of varying sample community compositions on the correlation between subsequent dominant species and environmental conditions, the species composition chord diagram reflects the cultivable endophytic fungi in the roots of *C. pilosula* under different spatiotemporal conditions (Fig. 6). As can be seen from the figure that the composition and proportion of cultivable endophytic fungi from the roots of different *C.pilosula* have significant differences at the genus classification level.

On the time scale, in spring, the abundance of endophytic fungi (9 genera) in MX area was the highest, followed by WY area (7 genera), and HC area (4 genera) the lowest. *A*s*pergillus* sp. (46.67%) and *Plectosphaerella* sp*.* (40.00%) were dominant genus in HC area, *Gylindrocarpon* sp*.* was its unique genera, and *Fusarium* sp. and *Phoma* sp*.* that existed in the other 5 regions have not been isolated from this region. *Aspergillus* sp. (37.50%) and *Phoma* sp*.* (25.00%) were dominant genera in LX area, *Paraphoma* sp. (31.25%), *Aspergillus* sp*.* and *Phoma* sp*.* (18.75%) were dominant genera in ZX area, *Dendryphion* sp. was endemic to the region, *Paraphoma* sp*.* (24.00%), *Aspergillus* sp*.* (20.00%) and *Phoma* sp*.* (16.00%) were core genus in MX area, and *Mucor* sp*.* and *Phaeosphaeriaceae* sp*.* were its unique genera, *Aspergillus* sp*.* (37.50%) and *Plectosphaerella* sp*.* (18.75%) were dominant genera in WY area. At the same time, *Epicoccum* sp*.* was endemic genera, *Fusarium* sp*.* (28.57%) and *Aspergillus* sp*.* (21.43%) were dominant genera in LT area.

In summer, LT area had the highest abundance of endophytic fungi (8 genera), followed by HC area and ZX area (7 genera), and WY area (2 genera) had the lowest abundance. *Aspergillus* sp*.* (40.00%), *Plectosphaerella* sp*.* (20.00%) and *Gylindrocarpon* sp*.* (15.00%*) are* dominant genera in HC area, *Gylindrocarpon* sp*.* and *Dactylonectria* sp*.* were its peculiar genera, and no other genera have been isolated in this area. *Fusarium* sp*.* existed in all regions. *Fusarium* sp*.* (33.33%), *Plectosphaerella* sp. (19.05%) and *Aspergillus* sp*.* (14.29%) were the dominant genera, and *Pyrenochaeta* sp. was its specific genera, *Fusa*rium sp*.* in ZX area. *Aspergillus* sp*., Plectosphaerella* sp. and *Phoma* sp*.* (19.35%) were the dominant genera, in MX area, *Aspergillus* sp. (37.50%), *Fusarium* sp. (25.00%) and *Penicillium* sp*.* (16.67%) were the dominant genera, *Mucor* sp*.* was its unique genus, only *Fusarium* sp*.* (78.57%) and *Aspergillus* sp*.* (21.43%) were isolated in WY area, *Aspergillus* sp*.* (41.67%) was the dominant genus in LT area, *Pleosporales* sp. and *Phaeosphaeriaceae* sp*.* And *Gaeumannomyces* sp*.* were its unique genera.

In autumn, the abundance of endophytic fungi (10 gen*era)* in HC area was the highest, and LT area (8genera) had the lowest abundance. *Fusarium* sp*.* (37.00%) and *Aspergillus* sp*.* (33.00%) were the dominant genera in HC area, while *Pyren*ochaeta s*p., Mortiere*lla sp. and *Trichoderma* sp*.* were endemic genera. In LX area, *Fusarium* sp*.* (30.50%), *Aspergillus* sp*.* and *Alternaria* sp*.*(25.42%) were the dominant genera, *Phoma* sp*.* and *Dactylonectria* sp. were endemic genera*, Aspergillus* sp*.* (27.27%) and *Fusarium* sp*.* (25.00%) were the dominant genera in ZX area, *Cladosporium* sp*.* was endemic genera, *Aspergillus* sp. (40.74%) and *Penicillum* sp*.* (25.93%) were dominant genera in MX area, *Gylindrocarpon* sp. was a unique genera, *Fusarium* sp*.*(36.96%) was dominant genera in WY area, *Fusarium* sp*.* (49.25%) and *Aspergillus* sp*.* (23.88%) were the dominant genera in LT area.

In winter, the abundance of endophytic fungi (7 genera) in HC area and WY area was the highest, and LT area (3genera) had the lowest abundance. In HC area, *Fusarium* sp. (*2*5.93%), *Alternaria* sp*., Penicillum* sp*., Plectosphaerella* sp. and *Muco*r sp. (14.82%) were the dominant genus, *Aureobasidium* sp*.* was an endemic genus, In LX area, *Fusarium* sp. (40.74%), *Aspergillus* sp. and *Alternaria* sp*.* (25.93%) were the dominant genus, *A*spergillus s*p.* (37.50%) and *Plectosphaerella* sp*.* (25.00%) were the dominant genera in ZX area, *Aspergillus* sp., *Alternaria* sp*.* and *Plectosphaerella* sp*.* (30.77%) was the dominant genera in MX area. In WY area, *Fusarium* sp. (40.00%) and *Aspergillus* sp*.* (25.00%) were dominant genera, *Mortierella* sp*.* was a unique genera. In LT area, *Fusarium* sp. (61.11%) and *Aspergillus* sp. (33.33%) was the dominant genus.

### Community composition specificity and consistency

**Fig. 7:**
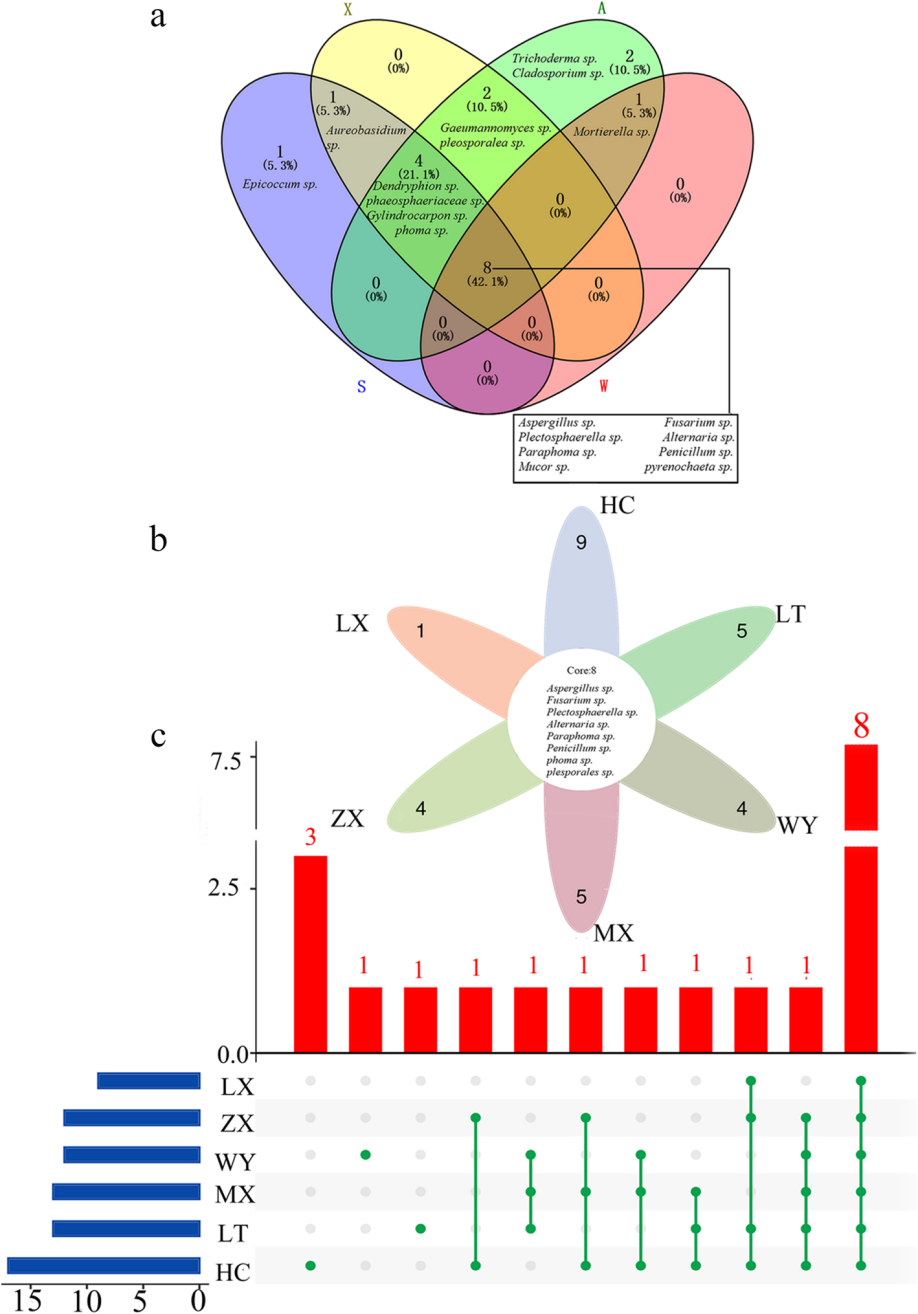
Venn diagram of common and endemic genera in four seasons and six regions. **a** Different colors represent different seasons, and the intersection represents their common genera. There are 8 genera in the four seasons. There are no endemic genera in summer and winter, but there are endemic genera in spring and autumn, one genus in spring and two genera in autumn. In addition, there is no common genus between winter and the other three seasons (**b**) The petal map of the common genera and endemic genera in the six regions, of which the total genera is 8 genera, HC region has the most endemic genera (9 genera) and LX region has the least (1 genera). **c** Visualization of Venn diagrams for common and unique genera in the six regions. The red bars represent the number of intersections between the groups, the blue bars represent the frequency of each group, and the green origin and connection lines represent the interaction between the groups

There are 8 genera of endophytic fungi in the four seasons (Fig. 7a), which indicated that they were the main genus that constructed the microbial community structure of the roots of *C. pilosula*. At the same time, the endemic genera in different seasons revealed that climate change might have a transformative effect on the rhizosphere microbial community structure of *C. pilosula*. For different regions, the same as the common genera in different areas, the six regions also shared 8 genera (Fig. 7b, c), which suggested that these genera were the central microbial communities in the roots of *C. pilosula* during different seasons. We analyzed the endemic genera in different regions found that HC region has the most endemic genera, which may be related to environmental factors (such as altitude) and sampling time in different regions. However, Comparing the main flora in different seasons and different regions, we found that their core flora was diverse. *Mucor* sp. and *pyrenochaeta* sp*.* was a genus shared by the four seasons, and *phoma* sp*.* and *pleosporalea* sp*.* was a regional genus, which further showed that seasonal changes had the most significant impact on the flora structure.

### Effect of soil physical and chemical properties on endophytic fungi diversity from *C. pilosula* roots in different plant locations at all seasons

**Fig. 8:**
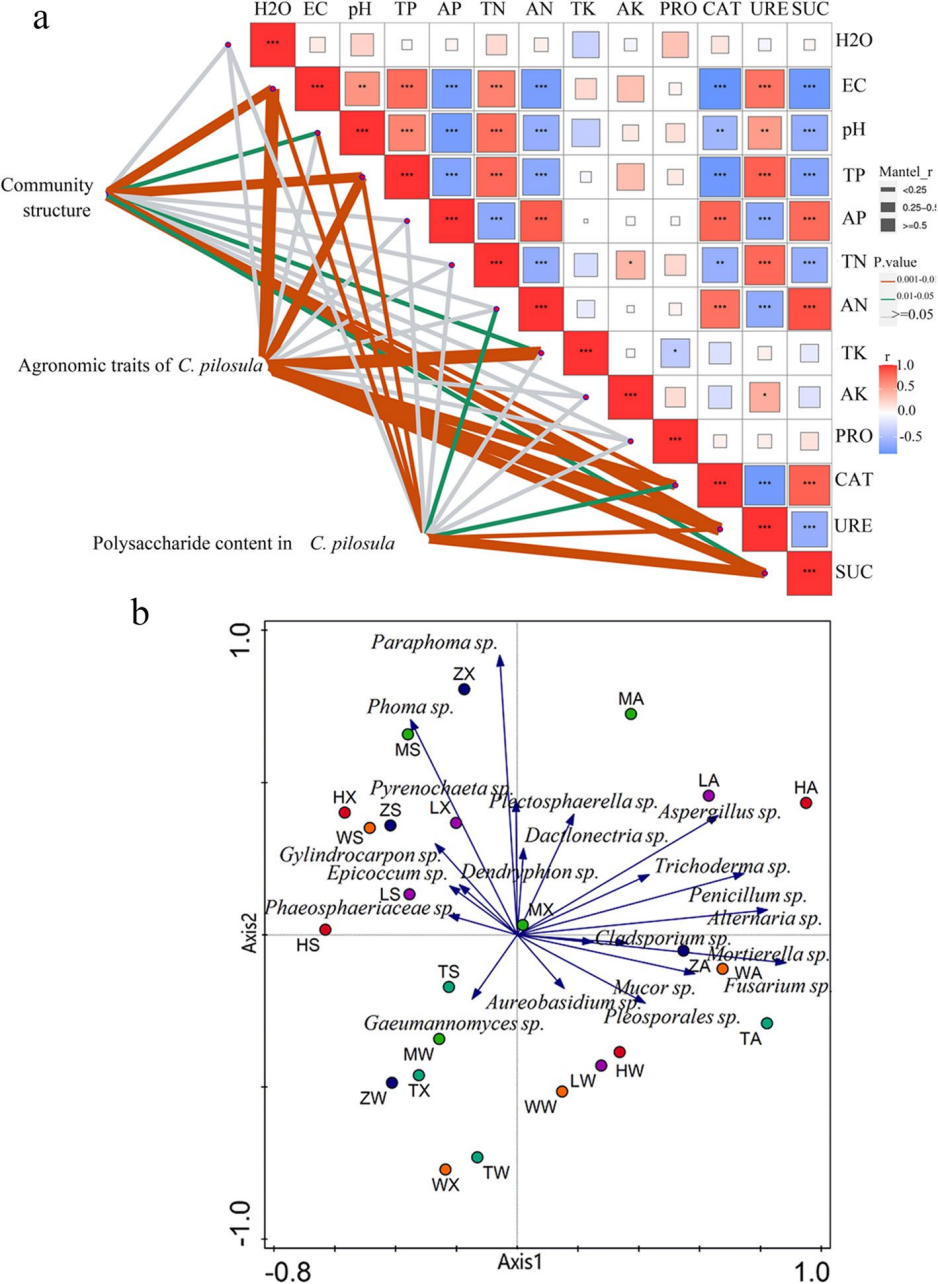
Correlation analysis. **a** MantelTest between soil physics and chemistry and species abundance. Different colored lines indicate different *P* values, Different color rectangles represent different correlations. The agronomic characters of *C. pilosula* were measured by root length (RL), root diameter (RD) and root weight (RW). polysaccharide content revealed the relationship between soil physics and chemistry and the accumulation of active components of *C. pilosula.* **b** Distribution of culturable endophytic fungi in roots of *C. pilosula* and their plots. Abbreviations in all figures have the following meanings. TN: Total nitrogen content in soil, AN: Content of available nitrogen in soil, TK: Total potassium content in soi, AK: Content of available potassium in soil, AP: Content of available phosphorus in soil, EC: Electroconductibility, H: Altitude, PRO: Protease, URE: Urease, CAT: Catalase, SUC: Sucrase

As shown in Fig. 8a, the results of Mantel Test correlation analysis show that soil physical and chemical properties EC, TP, TK, CAT, URE and SUC have significant effects on the microbial community (*P* < 0.05). EC, TP, TK, CAT, URE and SUC were significantly correlated with the agronomic traits of *C. pilosula* (*P* < 0.05). There was a significant correlation between soil physical and chemical properties (EC, pH, TP, AN, CAT, URE and SUC) and polysaccharide content in *C. pilosula* (*P* < 0.05). This revealed that soil nutrition and enzyme activity played an essential role in shaping the community in the root of *C. pilosula,* the growth and the accumulation of active substances of *C. pilosula.*

CANOCO 4.5 software was used to analyze the correlation of Endophytic Fungi of 20 genera isolated in four seasons in six main production areas of *C. pilosula* in Gansu Province, China. It can be seen from Fig. 8b that the sampling points in HC, LX and MX areas are distributed in the first quadrant in autumn. The sampling points of HC, LX, ZX, MX and WY were distributed in the second quadrant in spring and summer, The sampling points of LT in spring, summer and winter were the same as those of ZX, MX and WY in winter, The sampling points of ZX, WY and LT were the same as HC, LX and WY in autumn, and the sampling points were distributed in the fourth quadrant in winter, it showed that the diversity of culturable endophytic fungi in the roots of *C. pilosula* was different in different seasons. The distance between sampling points was relatively small in HC (summer), ZX and WY (spring), indicating that the composition of culturable endophytic fungi in the root of *C. pilosula* is similar. The distance between sampling points was relatively small in LT (summer), ZX and MX in winter, indicating that the composition of culturable endophytic fungi in *C. pilosula* root is similar. The distance between sampling points in HC, LX and WY is relatively small in winter, implying that the composition of culturable endophytic fungi in the roots of *C. pilosula* was similar. In general, the sampling points in four seasons in six main production areas of *C. pilosula* in Gansu Province were relatively scattered, suggesting that the community composition of culturable endophytic fungi in the roots of *C. pilosula* in different seasons was dynamic, which implied that seasonal variations was a factor driving the changes of endophytic fungi structure.

**Fig. 9:**
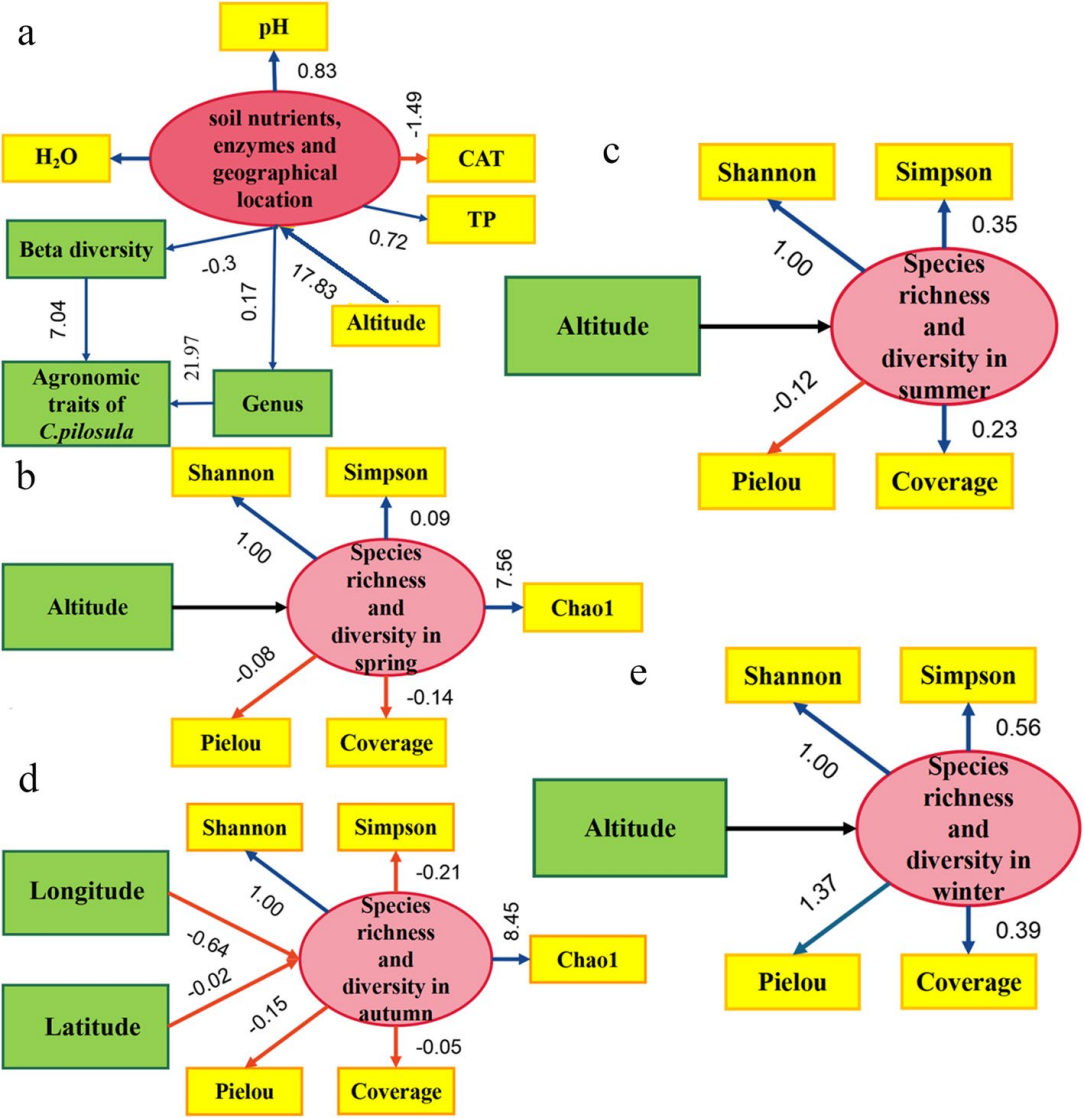
AMOS structural equation model explains the relationship among geographical locations, seasons and endophytic fungi diversity. **a** relationship with soil characterization, geographical locations, seasons and endophytic fungi diversity. **b**-**e** Correlation between geographical location and diversity index of culturable endophytic fungi in roots of *C. pilosula* in all seasons

**Fig. 10:**
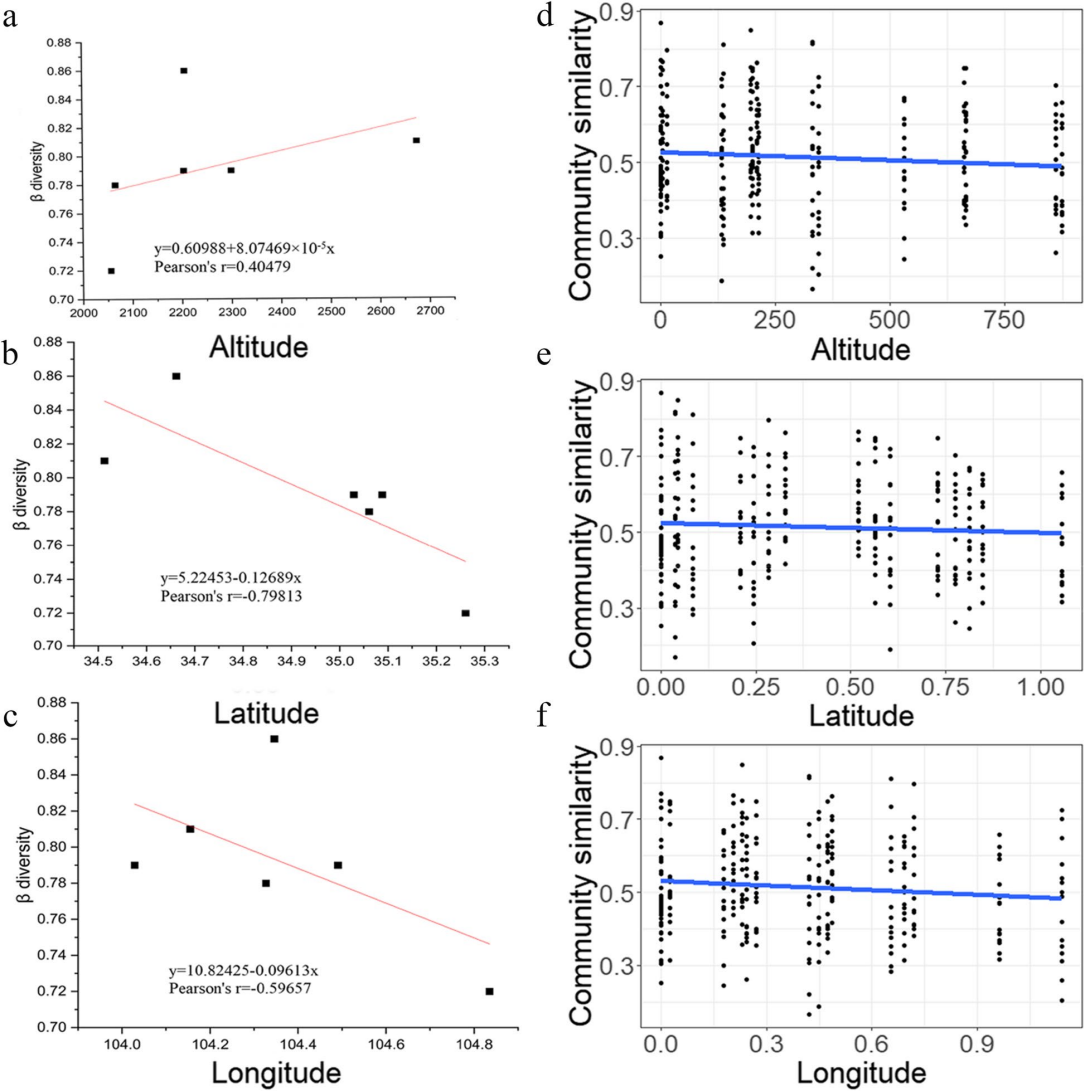
Correlation between altitude, longitude and latitude and flora diversity. The correlation between altitude, longitude, latitude and diversity is measured by the correlation coefficient r. When the value of r is between 0.0 and 0.2, there is a very weak correlation or no correlation. When the value of r is between 0.2 and 0.4, there is a weak correlation. The value of r is Between 0.4–0.6 indicates a moderate degree of correlation. when the r value is between 0.6–0.8, it means a strong correlation, and when *r* > 0.8, it indicates a powerful correlation. **a** The altitude of *C. pilosula* planting area is positively correlated with microbial community. **b** The latitude of *C*. *pilosula* planting area is negatively correlated with the diversity of flora. **c** The longitude of *C*. *pilosula* planting area is negatively correlated with the diversity of flora. **d**, **e** and **f** represent the distance attenuation model of altitude, latitude and longitude and microbial community diversity

Structural equation model shows the connection between soil nutrients, geographical locations, plant agronomic traits and culturable endophytic fungi (Fig. 9a). in general, microbial diversity was affected by altitude in geographical and drove by seasonal changes. It suggests that altitude plays a leading role in shaping microbial community structure in spring, summer and winter (Fig. 9b, c and e). microbial diversity in autumn is mainly affected by longitude and latitude (Fig. 9d). Distance attenuation model also shows that altitude is positively correlated with species diversity (*r* = 0.40479) while longitude and latitude are negatively correlated with species diversity. latitude and species diversity are strongly negative correlation (*r* = 0.79813), longitude and species diversity are moderately negatively correlated (*r* = 0.59657) species diversity (Fig. 10).

### Effect of soil physical and chemical properties, environmental distinction on endophytic fungi diversity from *C. pilosula* roots at all seasons

**Fig. 11:**
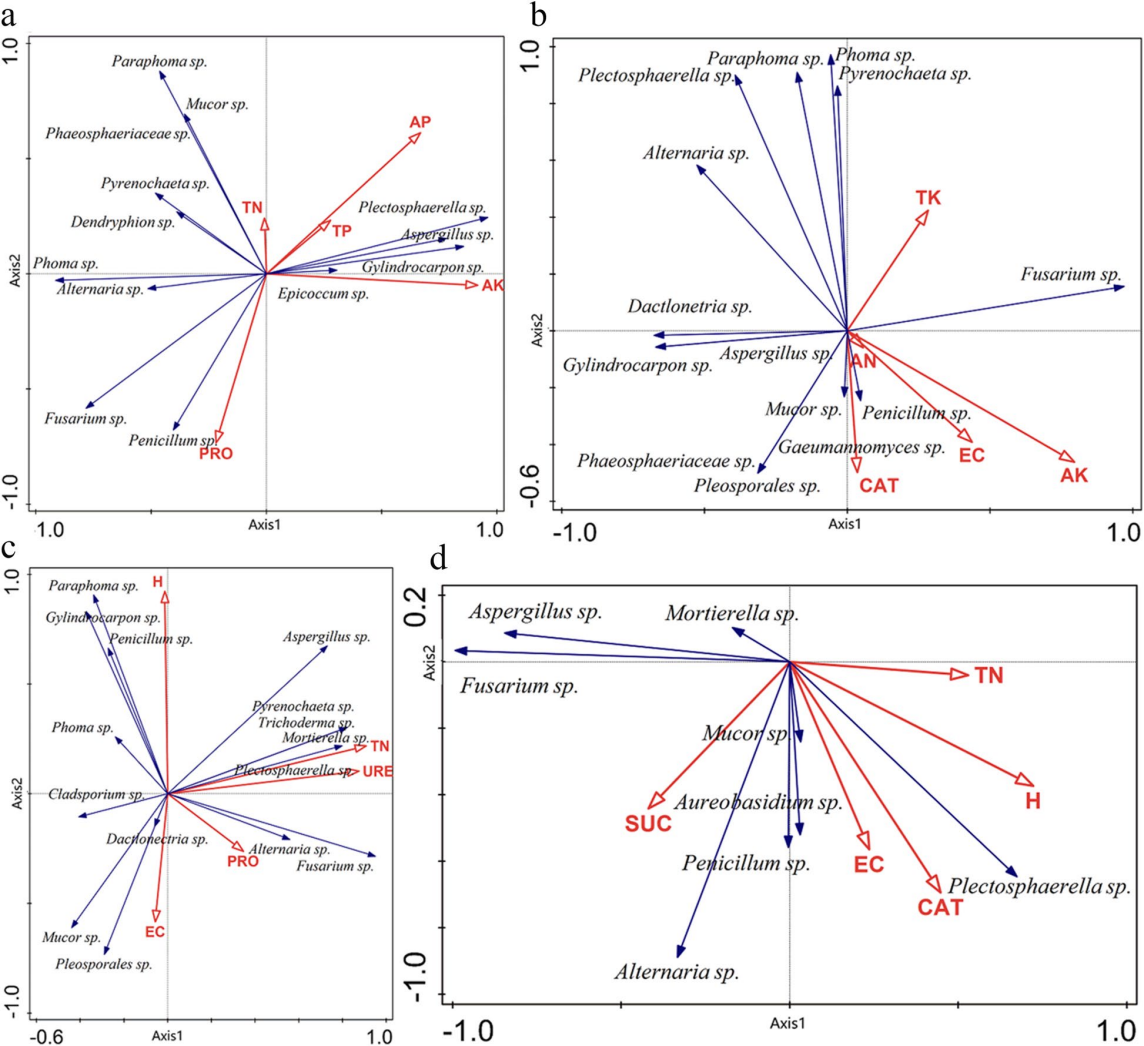
RDA of species and environmental factors. The blue lines represent different species, and the red lines represent different soil physical and chemical factors. The angle between the lines is an acute angle, which means a positive correlation between the two elements. If the angle is obtuse, it is a negative correlation. **a** Relationship between soil nutrients and microbial community in spring. **b** Relationship between soil nutrients and microbial community in summer. **c** Relationship between soil nutrients and microbial community in Autumn. **d** Relationship between soil nutrients, geographical pattern and microbial community in winter

The CANOCO 4.5 software screened 16 environmental factors in the six main producing areas of *C. pilosula* in Gansu Province and screened out 5 environmental factors that had a significant impact on the diversity of the cultivable endophytic fungal community in the roots of *C. pilosula.* (Fig. 11) Further analyze the contribution rate of these five dominant factors to the diversity of the fungal community.

In spring, the first axis of RDA analysis can explain 53.26% of all information, the second axis can explain 29.90%, and the cumulative amount of information presented is 83.16% (Fig. 11a). As can be seen that the first two axes can better reflect the relationship between fungal community diversity and environmental factors, and the first axis is the dominant axis. The main environmental factors playing a leading role in the diversity of the fungal community were TP, PRO, AK, TN and AP. The contribution rate of AK was 46.1%, which was the main factor driving the change of the endophytic fungal community. *Plectosphaerella* sp., *Aspergillus* sp., *Gylindrocarpon* sp., *Epicoccum* sp*.* were positively correlated with TN, AP, TP, and AK. Compared with the other three genera, environmental factors had little influence on *Gylindrocarpon* sp*., Paraphoma* sp*., Mucor* sp*.* had positive correlation with TN, TP, AP, *Pyrenochaeta* sp*., Dactylonectria* sp*.* showed positive correlation with TN, *Phoma* sp*., Alternaria* sp.*, Fusarium* sp*., Penicillium* sp*.* exhibited positive correlation with PRO. Compared with other 3 genera, the impact of environmental factors on *Alternaria* sp*.* was relatively small.

In summer, the first axis of RDA analysis can explain 48.77% of all information, the second axis can explain 29.81%, and the cumulative amount of information explained reached 78.58% (Fig. 11b). It can be seen that the first two axes can better reflect the relationship between fungal community diversity and environmental factors, The first axis is the dominant axis. The environmental factors playing a leading role in the diversity of the fungal community were mainly EC, TK, AK, AN and CAT. The contribution rate of AK was 38.0%, which was the main factor driving the change of the endophytic fungus community. *Fusarium* sp. was positively correlated with TK and AK, *Pyrenochaeta* sp*., Phoma* sp*., Paraphoma* sp*., Plectosphaerella* sp*., Alternaria* sp*.* were positively correlated with TK, *Dactylonectria* sp., *Gylindrocarpon* sp., *Aspergillus* sp*.* with TK, AK, AN, EC, and CAT were all negatively correlated, *Mucor* sp*.*, *Penicillium* sp., *Gaeumannomyces* sp*.*, *Phaeosphaeriaceae* sp*.*, *Pleosporales* sp*.* were positively correlated with AK, AN, EC, and CAT. Compared with the other three genera, environmental factors had little effect on *Mucor* sp*.* and *Penicillium* sp*.*

In Autumn, the first axis of RDA analysis can explain 66.72% of all information, the second axis can explain 22.39%, and the cumulative amount of information explained reaches 89.10% (Fig. 11c). It can be seen that the first two axes can better reflect the relationship between fungal community diversity and environmental factors, which were mainly determined by the first axis. The main environmental factors that played a leading role in the diversity of the fungal community were H, TN, URE, and PRO. The contribution rate of TN was 56.3%, which was the main factor driving the change of the endophytic fungus community. *Aspergillus* sp., *Pyrenochaeta* sp*.*, *Trichoderma* sp., *Mortierella* sp*.*, *Plectosphaerella* sp*.* were positively correlated with H, TN, URE, PRO, *Paraphoma* sp., *Gylindrocarpon* sp., *Penicillium* sp., *Phoma* sp*.* were positively correlated with H. Compared with the other three genera, environmental factors had relatively little influence on *Phoma* sp. *Cladosporium* sp. was positively correlated with EC. *Mucor sp.* and *Pleosporales* sp*.* were positively correlated with EC and PRO. *Alternaria* sp*.* and *Fusarium* sp*.* were positively correlated with TN, URE and PRO were positively correlated, and negatively correlated with H and EC.

In winter, the first axis of RDA analysis can explain 64.04% of all information, the second axis can explain 20.36%, and the cumulative amount of information explained reaches 84.40% (Fig. 11d). It can be seen that the first two axes can better reflect the relationship between fungal community diversity and environmental factors, which were mainly determined by the first axis. The main environmental factors that played a main role in the diversity of fungal communities were EC, TN, CAT, H and SUC. The contribution rate of H was 36.3%, which was the main factor driving community changes by endophytic fungi. *Mortierella* sp., *Aspergillus* sp., *Fusarium* sp*.* were positively correlated with SUC, and environmental factors had relatively little influence on *Mortierella* sp*.* Compared with the other two genera, *Alternaria* sp. was positively correlated with SUC, EC, CAT, and H. *Penicillium* sp., *Mucor* sp., *Aureobasidium* sp*.* were positively correlated with SUC, EC, CAT, H, and TN. Compared with the other two environmental factors, the influence of *Mucor* sp. was relatively slight, *Plectosphaerella* sp*.* and TN, H, CAT and EC were positively correlated, and negatively correlated with SUC.

In general, the analysis of the correlation between the cultivable endophytic fungi in the roots of *C. pilosula* and environmental factors in the four seasons found that the contribution rates of AP were 46.1% and 38.0% in spring and summer, respectively, which was the main factor driving the changes in the endophytic fungal community. In autumn, the contribution rate of TN reached 56.3%, which was the main factor driving the change of the endophytic fungus community. In winter, the contribution rate of altitude was 36.3%, that was the main factor driving the change of the endophytic fungus community. In addition, altitude had a significant effect on the shaping of community structure in autumn and winter, which was consistent with the previous results, which further revealed that the formation of culturable endophytic fungi community in *C. pilosula* root was affected by geographical environment.

## Discussion

Endophytic fungi are important symbionts in healthy plant tissues [31]. domestic and foreign research on *Codonopsis* mainly focuses on chemical composition, pharmacology and clinical application, and how to efficiently obtain abundant and diverse endophytic fungal resources and accurately identify them was the prerequisite for studying this hot spot. Studies have shown that soil physicochemical, endophytic fungi and their host plants had a significant interaction [32–37] The analysis of species diversity and community structure of culturable endophytic fungi in the rhizosphere of *C.pilosula* showed that seasonal changes and different altitudes drive the change of community structure. This was consistent with the results of previous studies [38, 39]. we found that *Trichoderma* sp. and *Cladosporium* sp. were the endemic genus in summer. *Epicoccum* sp. only exists in spring, which revealed seasonal changing may cause the structure of flora in all seasons. This result also has a similarity to previous research [40] which is found in the rhizosphere soils of *Argentina* (syn. *Potentilla*) anserina, on the Qinghai Plateau. The fungal abundance was affected by the sampling season [41–45]. Our study showed that the fungal abundance and community structure were significantly different in the four seasons, which were in dynamic change. Furthermore, most strains were isolated in autumn, and the isolation rate was the highest. This may be because the early phase of *C. pilosula* growth and development was mainly for vegetative growth, and there was the possibility of competition for nutrients with endophytic fungi, while the later stage was mainly for reproductive growth as nutrient reserves were relatively sufficient. In spring and summer had a low abundance and a small number, while in autumn and winter, the endophytic fungi were rich and have a large number. The physiological state of *C. pilosula* may be different in different seasons. there were specific differences in metabolism. In the months with suitable temperatures and sufficient sunshine, it can synthesize more nutrients. These changes were beneficial to the growth and reproduction of endophytic fungi.

The results of this experiment suggested that the diversity of endophytic fungi in *C. pilosula* was affected by the internal and external environment of *C. pilosula*, showing certain differences and specificity. This was consistent with the results of previous studies [39]. By studying the interaction between soil nutrients, geographical location, the diversity and community structure of culturable endophytic fungi in the roots of *C. pilosula*, we provided a basis for studying the factors affecting the accumulation of active components of *C. pilosula.*

## Conclusions

The results of this study shows that endophytic fungi diversity of *C. pilosula* is affected by temporal and spatial changes, which shows certain differences and specificities. And the time effect is more obvious. This suggests that climatic conditions may play a driving role in the growth and development of *C. pilosula*. The study of the diversity of endophytic fungi of *C. pilosula* not only provides a research basis and endophytic fungal resources for revealing its correlation with party ginseng in the future (such as growth promotion, synthesis and accumulation of active ingredients, etc.), but also contributes to the efficient and large-scale artificial cultivation and quality control of Gansu provincial medicinal plant in the future.

## Supplementary Information

**Additional file 1.**

## Data Availability

The datasets generated during and analyzed during the current study are available from the corresponding author on reasonable request.

## Abbreviations

CAT: Catalase
EC: Electroconductibility
URE: Urease
SUC: Sucrase
TN: Total nitrogen
AN: Available nitrogen
H: Altitude
PRO: Protease
RL: Root Length
RD: Root Diameter
RW: Root Weight
HC: Huichuan
LX: Longxi
ZX: Zhangxian
MX: Minxian
WY: Weiyuan
LT: Lintao
