# Supplementary material for: Culturable endophytic fungi community structure isolated from Codonopsis pilosula roots and effect of season and geographic location on their structures
Source: BMC Microbiol. 2023 May 15;23:132. doi: 10.1186/s12866-023-02848-3 (PMC10184406; doi:10.1186/s12866-023-02848-3)
Supplement: Supplementary file 1 — Additional file 1. [file 12866_2023_2848_MOESM1_ESM.docx]

**sTable 1** Sampling information of the *C. pilosula* from six main producing areas in Gansu Province, China

| No. | Scientific name | Code | Site of collection | Quarter | Collection time |
| --- | --- | --- | --- | --- | --- |
| 1 | Huichuan | HC | N 35°5'17'', E 104°1'43'', Altitude: 2300.5±5.2 m | Spring | 2017.3.3 |
| 2 | Longxi | LX | N 35°1'45'', E 104°29'26'', Altitude: 2204.0±5.2 m | Summer | 2017.6.2 |
| 3 | Zhangxian | ZX | N 34°39'42'', E 104°20'45'', Altitude: 2206.7±5.2 m | Autumn | 2017.8.29 |
| 4 | Minxian | MX | N 34°30'47'', E 104°9'18'', Altitude: 2674.7±5.2 m | Winter | 2017.11.28 |
| 5 | Weiyuan | WY | N 35°3'39'', E 104°19'37'', Altitude: 2065.7±5.2 m |  |  |
| 6 | Lintao | LT | N 35°15'38'', E 103°50'7'', Altitude: 2056.1±5.2 m |  |  |

**sTable 2** Diversity index calculated with the formula

| Diversity index | Formula |
| --- | --- |
| Shannon-Wiener（H） |  |
| Simpson（D） |  |
| Chao1（Schao1） |  |
| Coverage（C） |  |
| Pielou（J） |  |

**Note:** Species richness index: S=N, S is the total number of species in the plot. Schao1 is the actual number of genera obtained, n1 is the number of genera containing only one strain, n2 is the number of genera containing only two strains.


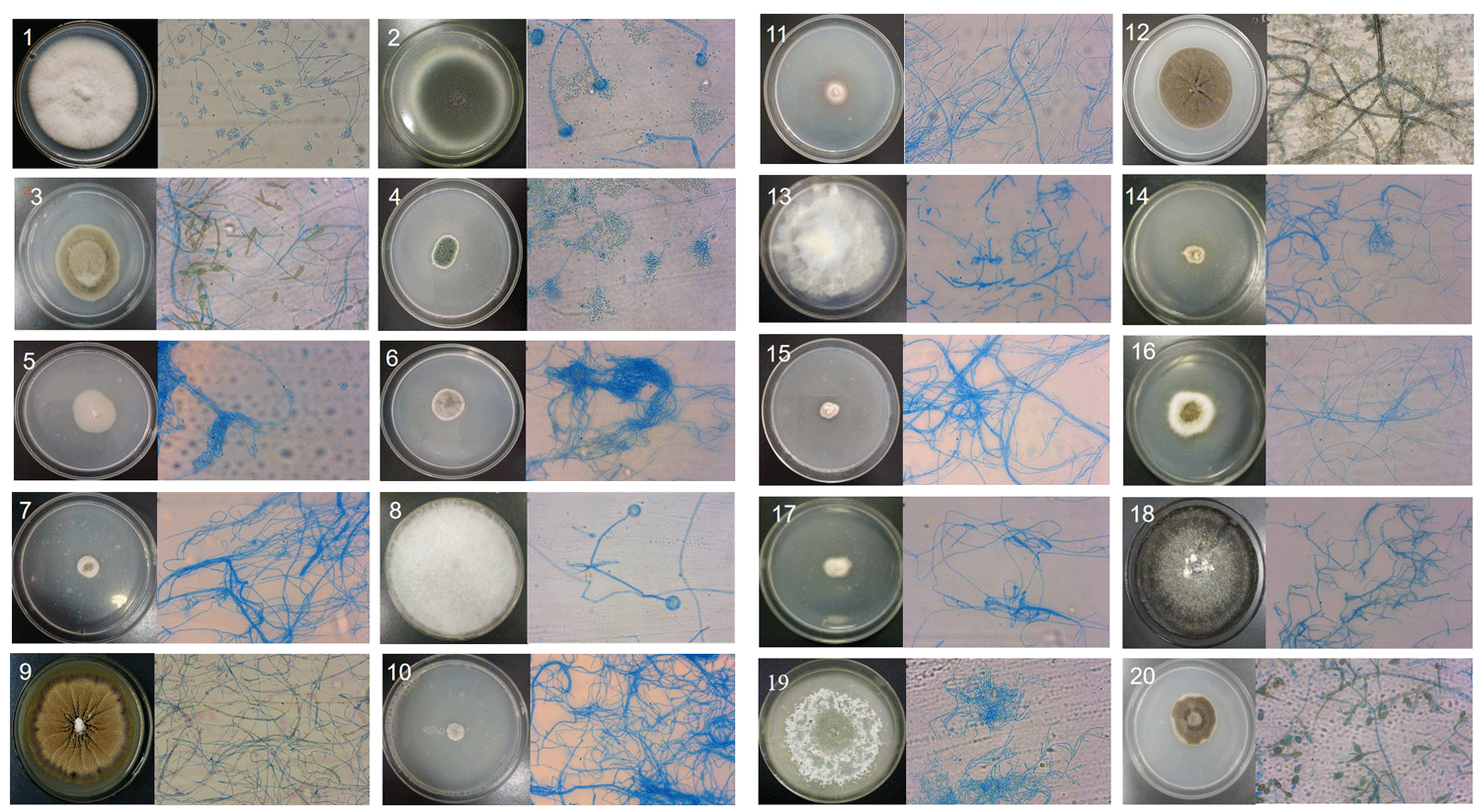


**sFig.1** Colony Morphology and Micrographics of 20 strains of Endophytic Fungi isolated from the *C. pilosula* Root
